# Warming at the population level: Effects on age structure, density, and generation cycles

**DOI:** 10.1002/ece3.4972

**Published:** 2019-03-28

**Authors:** Alice M. Laughton, Robert J. Knell

**Affiliations:** ^1^ School of Biological and Chemical Sciences Queen Mary University of London London UK

**Keywords:** climate change, competition, generation cycles, *Plodia interpunctella*, population, resource quality, warming

## Abstract

The impact of climate change on strongly age‐structured populations is poorly understood, despite the central role of temperature in determining developmental rates in ectotherms. Here we examine the effect of warming and its interactions with resource availability on the population dynamics of the pyralid moth *Plodia interpunctella,* populations of which normally show generation cycles, a consequence of strong and asymmetric age‐related competition. Warming by 3°C above the standard culture temperature led to substantial changes in population density, age structure, and population dynamics. Adult populations were some 50% larger in warmed populations, probably because the reduced fecundity associated with warming leads to reduced larval competition, allowing more larvae to develop to adulthood. Warming also interacted with resource availability to alter population dynamics, with the generation cycles typical of this species breaking down in the 30° populations when standard laboratory diet was provided but not when a reduced nutrient poor diet was used. Warming by 6° led to either rapid extinction or the persistence of populations at low densities for the duration of the experiment. We conclude that even moderate warming can have considerable effects on population structure and dynamics, potentially leading to complete changes in dynamics in some cases. These results are particularly relevant given the large number of economically important species that exhibit generation cycling, in many cases arising from similar mechanisms to those operating in *P. interpunctella*.

## 1 INTRODUCTION

Warming is the most obvious consequence of climate change, with climate prediction models currently forecasting changes in global temperatures of between 0.3 and 4.8°C by the end of this century (IPCC, 2014). Warming will have very obvious effects on populations of ectotherms because temperature influences almost all of the important processes during an animal's life history (Clarke, 2017). Given that developmental rates and the timings of transitions between life stages are among these processes, we can speculate that age‐structured populations are likely to be affected strongly by these temperature changes, but to date there has been little research on the effects of warming on such populations. Here, we investigate the effects of warming on one of the most dramatic effects associated with strong age‐structured effects in a population, namely generation cycles (Knell, 1998).

Generation cycles arise when an age‐structured population becomes more‐or‐less synchronized independently of any factors such as seasonal forcing. When this occurs, if one conspicuous life stage, usually the adults, is monitored the population appears to be undergoing cyclic dynamics with a period of approximately one generation. Although generation cycles are less well known than the famous multigeneration cycles exhibited by some animals, they are nonetheless important in many field systems, and arguably more important in economic terms. Populations of a wide variety of tropical insect species including many important pests exhibit generation cycles (Azerefegne, Solbreck, & Ives, 2001; Bigger, 1993; Bonsall, Hasan, & Nakamura, 2007; Godfray & Hassell, 1989; Hickel, Hickel, Souza, Vilela, & Miramontes, 2003; Nakamura, Hasant, Abbas, Godfray, & Bonsall, 2004; Wall, Howard, & Bindu, 2001; Yamanaka, Nelson, Uchimura, & Bjørnstad, 2012), as do a number of economically important fish species, including sockeye salmon, *Oncorhynchus nerka* (Guill, Drossel, Just, & Carmack, 2011), vendace *Coregonus albula* (Huusko & Hyvärinen, 2005; Marjomäki, Urpanen, & Karjalainen, 2014), and possibly bluefin tuna *Thunnus thynnus* (Ravier & Fromentin, 2001). *Daphnia* populations are also known to exhibit generation cycles in both the laboratory and the field (McCauley & Murdoch, 1987).

Generation cycles can be generated by intraspecific competition, especially when there is asymmetric competition between life‐history stages (Briggs, Sait, Begon, Thompson, & Godfray, 2000; Gurney & Nisbet, 1985; Gurney, Nisbet, & Lawton, 1983), and the cycles observed in a number of field systems have been attributed to this mechanism (e.g., Nakamura et al., 2004; Marjomäki et al., 2014). Generation cycles can also be generated by interactions with a natural enemy such as a parasitoid, especially when the reproductive phase of the host's life cycle is relatively short and the generation time of the natural enemy is roughly one‐half or one‐and‐a‐half times that of the host (Godfray & Hassell, 1987; Knell, 1998; Reeve, Cronin, & Strong, 1994).

For the present study, we used replicated, multigeneration laboratory mesocosms to test the effects of warming on populations of the pyralid lepidopteran *Plodia interpunctella*. *P. interpunctella *is a model insect system which has been extensively studied by population ecologists to help us understand phenomena such as the effect of age structure and asymmetric competition on population dynamics (Bjørnstad et al., 1998; Briggs et al., 2000; Gurney et al., 1983) and the long‐term dynamics and evolution of populations with natural enemies present (Begon, Sait, & Thompson, 1996; Boots, Childs, Reuman, & Mealor, 2009; Boots & Mealor, 2007; Knell, Begon, & Thompson, 1998a; Sait, Begon, & Thompson, 1994; Sait, Liu, Thompson, Godfray, & Begon, 2000). Laboratory populations of *P. interpunctella *typically exhibit generation cycles (Sait et al., 1994) which are believed to be a consequence of the strong asymmetric competition between larval stages: Larger 4th and 5th instar larvae are aggressive and will not only compete for food with the younger larvae but also cannibalize them along with eggs and sometimes pupae (Cameron, Wearing, Rohani, & Sait, 2007; Reed, 1998). This strong asymmetric interaction means that the age structure of populations of *P. interpunctella* becomes synchronized, causing generation–length cycles (Briggs et al., 2000; Sait et al., 1994). Both statistical modeling of population time series (Bjørnstad et al., 1998) and mathematical population modeling (Briggs et al., 2000) support this and emphasize the importance of the asymmetry in competition between older and younger larvae in driving these generation cycles (Bjørnstad et al., 1998).

Predicting the effects of increased temperature on populations exhibiting generation cycles is not straightforward. Grover, McKee, Young, Godfray, and Turchin (2000) found no consistent effect of warming on cycling in *Daphnia*, but the authors were not able to tell whether the cycles in their populations were generation cycles or multigeneration cycles. In the case of *P. interpunctella, *there has been a considerable amount of research on the life‐history effects of temperature, summarized in Table 1. Work on the strain used in these experiments found that increasing temperature from 27°C (the standard laboratory rearing temperature) to 30°C reduces the egg‐to‐eclosion period by 2–3 days and adult lifespan by 2.5 days, with 50% of unmated females surviving for 12.5 days at 27°C and 10 days at 30°C (Laughton, O'Connor, & Knell, 2017), suggesting that generation cycles should become shorter with increasing temperature. This assumes that other temperature effects on life‐history characters in *P. interpunctella* do not influence the behavior of the populations, however, and it is possible that changes in factors like recruitment could override this: Lifetime fecundity of females is reduced by 20%–25% by an increase from 27 to 30°C (Laughton et al., 2017). Increasing temperature has also been found to have a pronounced effect on egg‐to‐adult survival in *P. interpunctella*, with the proportion of eggs leading to eclosing adults changing from roughly 60% at 27°C to between 20% and 30% at 33°C (Parrett & Knell, 2018). These reductions in fecundity and survival could potentially weaken intraspecific competition, thereby reducing the tendency of the populations to cycle.

**Table 1:** Effects of changing temperature on life‐history traits for *Plodia interpunctella*

Measure	Effect	References
Developmental period	Fastest at 32° (approx. 20 days), slower at 35° (25 days) and at 28 and 25° (23 days and 28 days respectively)	Johnson, Wofford, and Whitehand (1992)
Developmental period	Decreases from roughly 170 days at 17° to roughly 35 days at 32° depending on food	Na and Ryoo (2000)
**Egg‐to‐pupation period**	**Decreases as temperature increases from 20**°** to 27**°** but little change from 27 to 30**°	Laughton et al. (2017)
**Duration of pupal period**	**Decreases from roughly 18 days at 20**°** to roughly 6 days at 30**°	Laughton et al. (2017)
Lifespan	Declines linearly with increasing temperature between 20 and 35°C	Arbogast (2007)
**Adult lifespan females**	**Decreases from roughly 18 days at 20**°** to between 6 (mated) and 9 (unmated) days at 30**°	Laughton et al. (2017)
Fecundity	Maximized between 25 and 30°C	Arbogast (2007)
**Fecundity schedule**	**At 20 and 24**°** females lay consistent numbers of eggs per day throughout their lifespan. At 26, 28, and 30**°** females lay most of their eggs in the first few days of adulthood**	Laughton et al. (2017)
**Fecundity**	**Roughly constant from 27**°** to 31**°** but declines as the temperature increases further**	Parrett and Knell (2018)
Egg hatching	Lower at 25° (92%) but no significant difference between 28, 32 and 35° (all roughly 97%)	Johnson et al. (1992)
Egg‐to‐adult survival	No significant effect of increasing temperature from 25 to 35°	Johnson et al. (1992)
Egg‐to‐adult survival	Increases from 17° to 25° and then either plateaus or increase as temperature increases to 32°	Na and Ryoo (2000)
**Egg‐to‐adult survival**	**Decreases slightly from 29 to 31**°**, drops sharply at 33**°** and zero at 35**°	Parrett and Knell (2018)
Lifetime reproductive success	No significant difference between 25 and 32° but zero at 35°	Johnson et al. (1992)

Note

Bold text indicates that the measurements were made on the same strain of *P. interpunctella* as that used in the present study.

A further potential influence of global change that is likely to interact with warming is resource availability. As the climate changes, so will the availability and quality of resources such as food (Tylianakis, Didham, Bascompte, & Wardle, 2008), and so populations experiencing warming will also experience multiple other changes to their environment. Food quality is known to affect population dynamics in *P. interpunctella*. Some studies used a very rich diet based on commercial baby food fortified with yeast, glycerol, and honey, and these produce clear and well‐defined cycles (Boots et al., 2009; Sait et al., 1994). Other work has used a less rich diet based on bran, and *P. interpunctella* populations reared on this show less well‐defined cycles or no cycles at all, probably because of a reduction in population density in the latter populations (Knell et al., 1998a; McVean, Sait, Thompson, & Begon, 2002a). Because of this importance of resource quality in *P. interpunctella*, here we manipulate resource availability as well as temperature, with half of our populations being raised on standard laboratory diet and half on a low nutritional content diet.

Given the previously described effects of poor diet on *P. interpunctella* population dynamics, we predict that generation cycles will be less likely in populations given poorer quality diets. How poor quality food might interact with temperature to affect *P. interpunctella *populations is not clear, but given the importance of resource‐based competition in this system and the effects of temperature on fecundity and survival, it is possible that these factors will interact with food quality to alter population dynamics.

## 2 METHODS

### 2.1 Study animals

*Plodia interpunctella*, a pyralid moth, is a globally distributed pest of stored foods, chiefly infesting stored grains, pulses, and nuts (Mohandass, Arthur, Zhu, & Throne, 2007) but also attacking a range of other foodstuffs from dried vegetables (Na & Ryoo, 2000) to chocolate (Olsson, Anderbrant, & Löfstedt, 2005) and instant noodles (Murata, Imamura, & Miyanoshita, 2006). *P. interpunctella* is easy to culture and to maintain in multigeneration population cages in the laboratory, and it has been used to produce a series of important and detailed insights into the ecology and dynamics of single populations (Bjørnstad et al., 1998; Briggs et al., 2000; Cameron et al., 2007; McVean et al., 2002a), host–pathogen systems (Boots et al., 2009; Knell et al., 1998a; Knell, Begon, & Thompson, 1998b; Sait et al., 1994), and systems with multiple natural enemies (Begon et al., 1996; Bjørnstad, Sait, Stenseth, Thompson, & Begon, 2001).

An outbred stock culture of *P. interpunctella* was established in 2013 by combining three stock lines: one maintained at the University of Liverpool for at least a decade, one collected in Perth, Australia in 2001, and a commercially available line maintained at Fera Science Limited (http://fera.co.uk/). The outbred line was allowed to establish for at least 10 generations in standard laboratory conditions of 27°C, 12L:12D light cycle, *ad lib*. standard diet of 10:1:1 ratio of organic wheat bran (Mount Pleasant Mill, Lincolnshire), brewer's yeast and glycerol, before being used for experiments.

### 2.2 Incubators

In order to produce a properly replicated study, separate incubators were built, one for each population. These consisted of an expanded polystyrene box (40 × 30 cm base and 22 cm height) containing a vivarium heat mat connected to a thermostat. Each heat mat was covered with a layer of vermiculite to ensure convective rather than radiative heat transfer, and each incubator was fitted with a standard PC fan to ensure good circulation. Population cages were propped on supports approximately 5 cm above the heat mats, and a window was cut in the front of each incubator and double glazed with acrylic sheet to allow light in. All incubators were kept in a controlled temperature (CT) room at 26°C and were repositioned within the CT room every few weeks to ensure no effects of shelf location on population dynamics. iButton Thermochron data loggers (Maxim Integrated, San Jose, California www.maximintegrated.com) were used to monitor temperature in each incubator.

### 2.3 Experimental design

Eighteen populations were established, each using 100 mixed sex (assumed to be 50:50 based on preliminary surveys) larvae from the outbred line. Populations were kept in plastic containers (25 × 25 cm base and 15 cm height) in individual incubators at 27°C, 30°C, or 33°C ± 0.5°C, and on one of two diets: either a standard laboratory diet (as above, 10:1:1 ratio of organic wheat bran, brewer's yeast, and glycerol), or a nutritionally stressful “poor” diet (replacing 50% of the wheat bran with methylcellulose, an indigestible bulking agent). Treatment combinations followed a fully factored design, giving three replicate populations for each temperature × diet combination. All populations were exposed to the same 12L:12D light cycle.

The base of each population container was divided into a grid of six large and three small sections. After an initial undisturbed 2‐week establishment period, each week on rotation one large and one small section of diet was removed and replaced with the appropriate fresh diet. The extracted sections contained both live larvae and dead adults. Each small section of diet (3 weeks old) was sorted, all larvae were removed, and the number, instar (3rd, 4th, or 5th), and sex (testes become visible at 4th and 5th instar) recorded. 1st and 2nd instar larvae were not counted because their extremely small size made it impossible to obtain accurate counts. After sorting, larvae were returned to their original populations. Dead adults were collected from the entire population container, including those from both the small and large sections, and all were sexed and counted before being discarded. The populations were allowed to run continuously, with larval data collected for 62 weeks, and dead adult data collected for 82 weeks, approximately 14 generations.

### 2.4 Data analysis

All analyses of population data were carried out using a truncated time series, with the first 10 weeks removed so as to avoid bias from transients associated with the initial setup of the populations. Quantitative population‐level measures such as mean population sizes were compared using linear models, with nonsignificant terms removed to produce a minimal adequate model (Zuur, Ieno, & Walker, 2009), whereas qualitative measures such as whether generation cycles were present or not were compared using Fisher's exact test.

The dynamics of each population were explored by examining spectral densities (Shumway & Stoffer, 2017), complemented by wavelet analysis (Cazelles et al., 2008). Spectral analysis is a technique which decomposes a time series of data into its component frequencies, and the relative importance, or density, of these frequencies is then displayed on a periodogram, with peaks in the spectral density indicating periodicities in the data. The population data were square‐root transformed prior to this analysis in order to normalize the errors, and approximate statistical significance thresholds for these analyses were calculated as the 95th quantile for spectral densities of 10,000 replicates of white noise drawn from a negative binomial distribution with the mean and shape parameter equal to those for the time series in question.

Wavelet analysis also looks for periodicities in the data but does not assume “stationarity” in the time series, meaning that if the dynamics change over the course of a time series this can be visualized, so if, for example, there are cycles in a time series that change period this will be detected and visualized. Because the long‐period cycles in these populations have wavelengths which are greater than 25% of the total length of the time series, these were removed from the data by smoothing with a loess function of span 0.4 prior to analysis. Wavelet transformations were carried out using the WaveletComp package (Roesch & Schmidbauer, 2018) using a Morlet wavelet and plotted with 5% significance contours calculated using the default (white noise) options.

All analyses were carried out in R v. 3.5.0 (R Development Core Team, 2013). Full analysis details and the corresponding R code are available in Supporting information Appendix S1.

## 3 RESULTS

**Figure 1:**
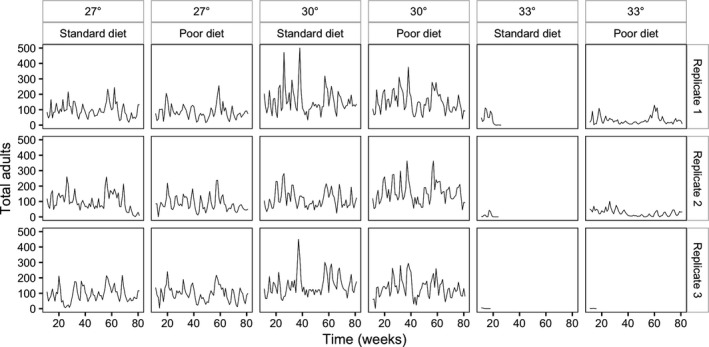
Time series of weekly dead adult counts for all replicate population containers from week 10 to week 82. All populations at 27 and 30°C persisted but four of the populations at 33°C became extinct rapidly, whereas the remaining two survived for the duration of the experiment

Figure 1 shows the time series for each population. All populations at 27 and 30°C persisted for the entire 82 weeks. All of the 33°C populations from the standard diet treatment went extinct in a few generations, but two of the 33°C populations from the poor diet treatment persisted for the full period. The populations that went extinct all did so in less than 6 months, so the high temperature populations either went extinct rapidly or persisted for a long time.

**Figure 2:**
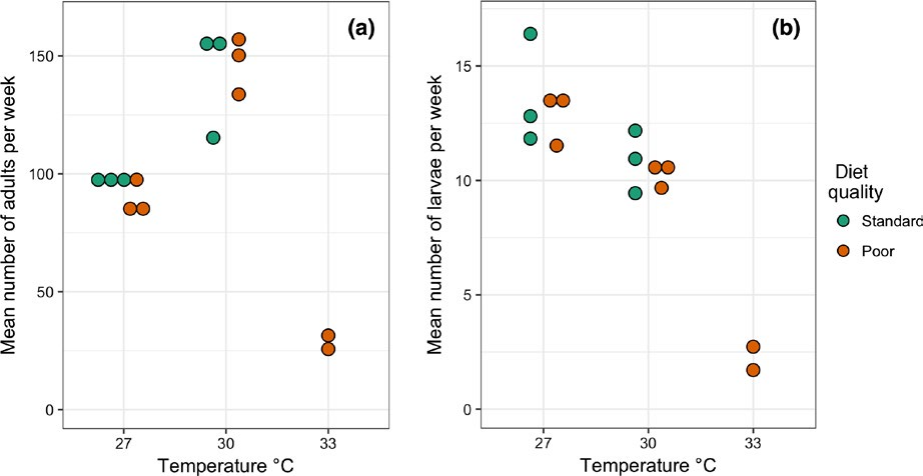
Mean population sizes of adults (a) and larvae (b) for each temperature and diet treatment. Adult populations were greatest in the 30°C populations, whereas larval populations were greatest in the 27°C cages. Diet quality had little effect on population sizes. Note that adult counts are for the whole cage, whereas larval counts are only for one small section of each population container, so values for adults and larvae are not directly comparable. Larval counts are for 3rd to 5th instar larvae only, 1st and 2nd instar larvae being too small to count reliably

Figure 2a shows the mean population sizes (as weekly counts of dead adults) for each population. There was a highly significant effect of temperature on population size (*F*_2,9_ = 43.6, *p* = 0.00002), with the 30°C treatments having the highest numbers of adult moths and the two 33°C populations which persisted having the lowest. There was no significant effect of diet quality or the interaction between diet quality and temperature. By contrast with the data for adults, there were fewer larvae in the 30°C treatment than in the 27°C one (Figure 2b), and the two 33°C treatments which persisted had very low larval numbers. As with the adult population data, there was no effect of diet quality on larval population sizes, but a highly significant effect of temperature (*F*_2,11_ = 48.4, *p* < 0.00001).

Visual inspection of Figure 1 also suggests that the 30°C populations are more variable than the 27°C ones, but this is an artifact of their larger population sizes. If the coefficients of variance (CVs) are calculated for each population (see Figure 6 in Appendix 1), the values for the 30°C populations are in fact significantly lower than those for the 27°C populations, and the 33°C populations have considerably higher CVs than either of the two cooler temperatures (mean CV 27°C = 0.531 ± 0.041 [95% CI], 30°C = 0.476 ± 0.033, 33°C = 0.829 ± 0.25, linear model, main effect of temperature *F*_2,11_ = 79.0, *p* < 0.0001). Diet quality had no statistically significant effect on the degree of variability of the populations.

**Figure 3:**
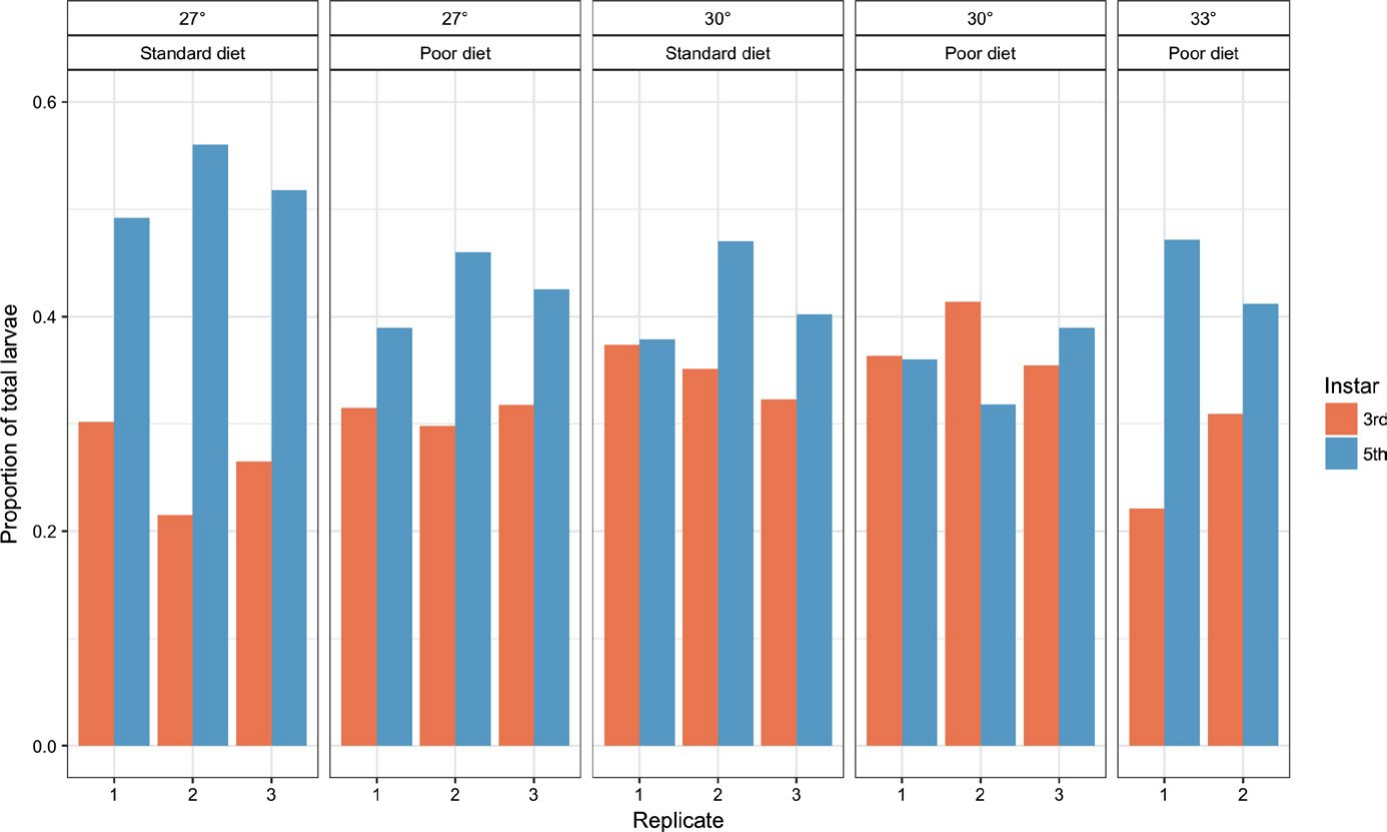
Warming to 30°C and reducing the quality of the available diet both make the larval age structure more even, with proportionally fewer 5th instar larvae and proportionally more younger larvae. 3rd, 4th, and 5th instar larvae were recorded, but 4th instars are not shown for clarity. 1st and 2nd instar larvae are too small to count reliably. The effect seems to be somewhat reversed in the two populations at 33°C which persisted

Figure 3 shows the time‐averaged age structures for the larval populations. Considering only the 27 and 30°C populations, there was a significant interaction between temperature and diet on the proportion of 5th instar larvae such that overall the age structure of the population became more even (*F*_1,7_ = 6.91, *p* = 0.034). Either increasing the temperature from 27 to 30°C or changing from standard to poor diet quality reduced the proportion of 5th instar larvae by 6%–8%, but when the temperature was 30°C there was little effect of changing from standard to poor diet on age structure. Both of these treatments therefore lead to a more even distribution of larval age classes. Interestingly, this trend did not appear to continue for the two 33°C populations which persisted. Despite having the highest temperature and poor diet, the age structure of both larval populations was intermediate, with proportionally more 5th instar larvae than any of the 30°C and poor diet populations, but proportionally fewer than the 27°C and standard diet populations.

**Figure 4:**
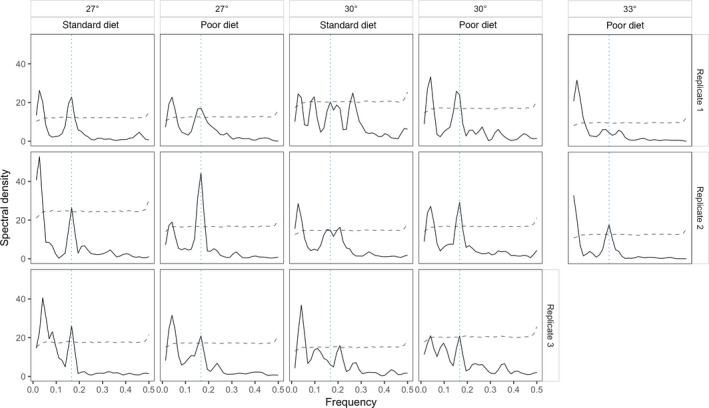
Spectral densities for each of the time series from populations which persisted for the full experiment. Peaks indicate periodicity in the time series, and frequency (the x‐axis) is equal to 1/the wavelength in weeks. Peaks, which cross the dashed line, are statistically significant. All of the populations at 27°C and the “poor diet” populations at 30°C show strong peaks at a wavelength of 6 weeks (indicated by the dotted vertical lines), indicating regular cycles of one generation in length—although one of these, that for replicate 3 of the 30°C poor diet populations is only slightly over the significance threshold. The 30°C populations given standard diet, by contrast, all show multiple, marginally significant peaks with little consistency. One 33°C population shows generation cycles, but the other does not. All time series also show peaks at frequencies close to zero, which arise from the multigeneration cycles which are apparent in the time series in addition to the generation cycles

The spectral density plots (Figure 4) show that there are two important periodicities in the data: first a long‐period cycle of between 25 and 35 weeks, as indicated by the peak on the left hand side of the graphs, and second, in some populations, short‐period generation cycles of 6 weeks overlaid onto the longer term cycles. This is shown by the peak that in most cases aligns with the vertical dotted line, which indicates a frequency of 1/6 on each graph. These longer period cycles varied between populations, with some being approximately 36 weeks and some shorter (approximately 24 weeks). Note that because of the length of these cycles the spectral analysis will only fit peaks corresponding to periods of 72, 36, 24, 18, etc., so the precise lengths of the long‐period cycles are hard to estimate.

Regarding the shorter period cycles, the spectral densities reveal strong and statistically significant peaks corresponding to a period of 6 weeks in all of the 27°C populations—generation cycles. The dynamics of the poor diet treatments are similar at 27 and 30°C, with generation cycles at a period of 6 weeks, although the peak for replicate 3 at 30°C is only just statistically significant. The standard diet populations also exhibited generation cycles at 27°C, but at 30°C all three populations gave complex spectra with multiple, marginally significant peaks and little indication of generation cycles. This pattern, with all of the 27 and 30°C populations showing six‐week generation cycles except for the 30°C standard diet treatment, is statistically significant (Fisher's exact test, *p* = 0.018). Of the 33°C populations which persisted, one had little indication of generation–length cycles, whereas the other had a strong peak at a frequency corresponding to a 6‐week period.

**Figure 5:**
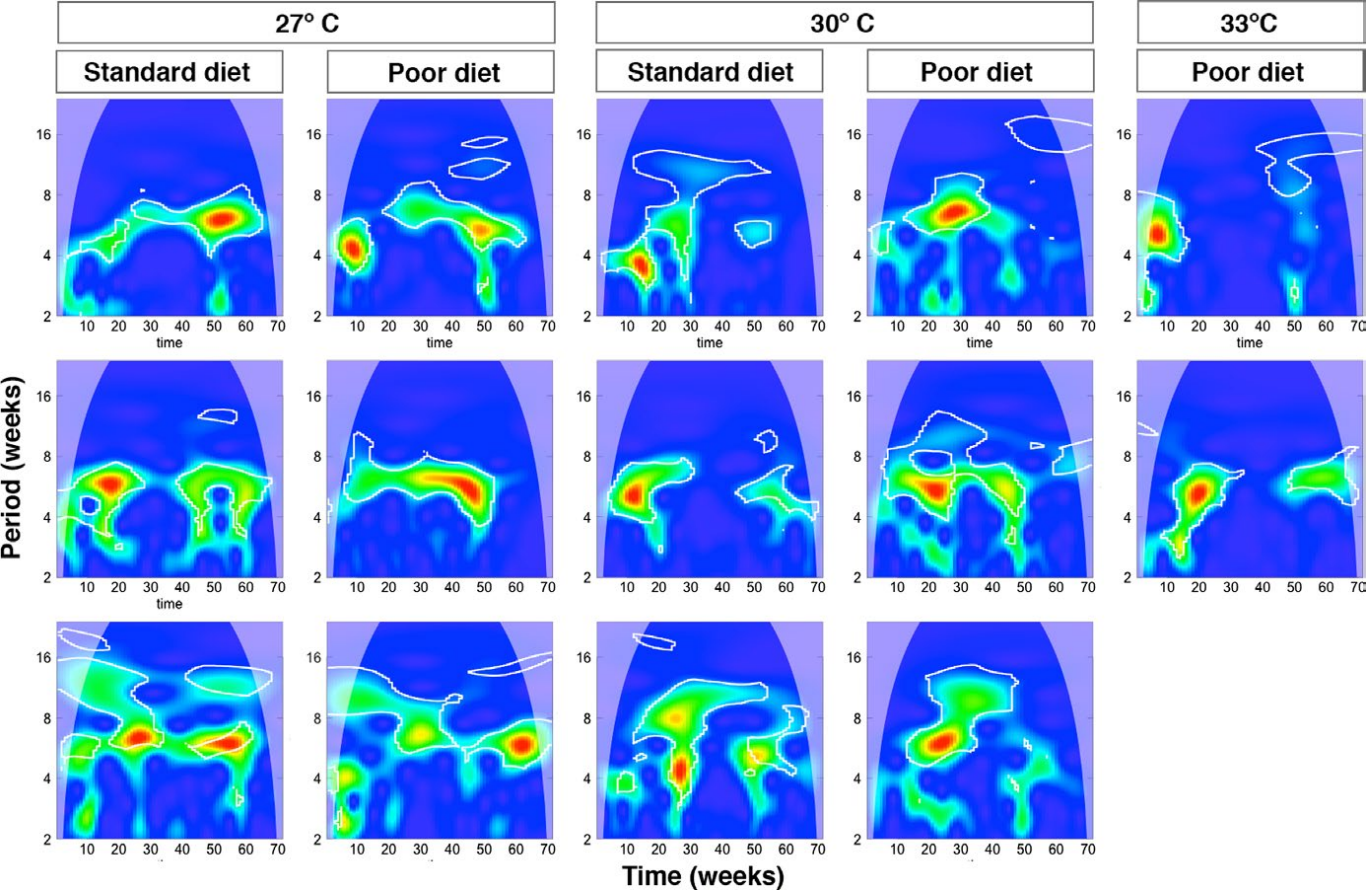
Wavelet power spectra of population time series. The x‐axis gives time in weeks, and the y‐axis indicates the period. The power is indicated by color, with blue indicating low power, whereas green, yellow, and red show increasing power, and the paler regions at the left and right hand sides of the plots show the regions where effects associated with the beginning and end of the time series reduce certainty. The time series identified by spectral analysis (Figure 4) as showing 6‐week generation cycles, including all of the 27°C populations, the 30°C poor diet populations, and the second replicate of the 33°C poor diet treatment all show areas of high power at a period of 6 weeks, with those with particularly strong 6‐week peaks in the spectral analysis showing “ridges” of power across the time series, indicating that generation cycling is occurring for most of the period that the populations were maintained. The 30°C populations maintained on standard diet, by contrast, show brief periods of regularity at a variety of periods, and only the second one shows any peak of power near the 6‐week periodicity found in the other time series. The 33°C population which does not show generation cycles (the first one) has a very brief period of cycling at the start of the time series after which there is no real periodicity. Populations which became extinct during the experiment are not shown

**Figure 6:**
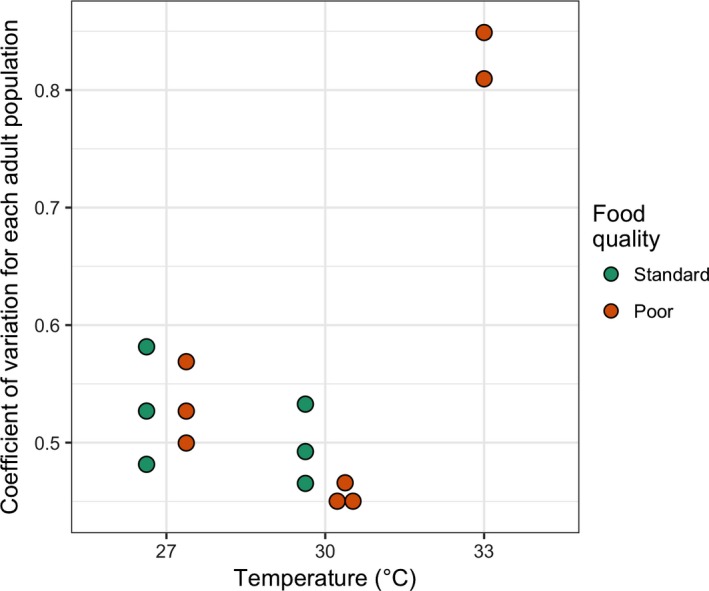
Coefficients of variation for each population

These results are supported by wavelet analysis (Cazelles et al., 2008) which finds strong evidence for 6‐week cycles in the 27°C populations and the 30°C populations reared on poor diet (Figure 5)—there is a clear a “ridge” of highly significant wavelet power corresponding to a period of roughly 6 weeks extending across most of the time series. This was clearer in some (e.g., 27°, poor diet, replicate 2) than in others (e.g., 27°, standard diet, replicate 1) and in none of the populations did the ridge persist for the entire period—all exhibited some transient losses of periodicity at various points. Of the 30°C populations, those on poor diet all showed similar behavior to the 27°C populations, although the third replicate in this group only showed evidence for strong 6‐week cycles for a relatively brief period (as noted above, this replicate had a weak and only fractionally significant 6‐week peak in the spectral analysis). The three 30°C populations given standard diet, however, show transient periods with 4‐, 6‐, and 8‐week periodicities (especially visible in populations 3 and 15), as well as periods with no underlying periodicity (e.g., population 3 after about 45 weeks), and little evidence for sustained generation length cycles.

## 4 DISCUSSION

Most attempts to understand how warming will affect life on Earth have focussed on species persistence and extinction, and how this will relate to community and ecosystem functioning. Here we take an alternative approach and look in detail at the population biology of a single species system. We find that even when a population persists and is apparently healthy, its ecology can be significantly altered by even a moderate degree of warming. An increase of 3°C from the standard laboratory rearing temperature for *Plodia interpunctella* leads to substantial changes in the moth's population biology. Adult populations are larger but larval populations are smaller, and the age structure of the larval populations changes, with a more even distribution of age classes in the warmer populations. Temperature interacts with resource availability to determine the population dynamics of the system, with the generation cycles which are typical of *P. interpunctella* populations seen in all cases at 27°C and in 30°C populations with poor diet, but not in the 30°C populations given standard diet. Overall, the 30°C populations were less variable than the 27°C ones.

Many of these changes with warming are likely to be related to changes in the strong competition between larvae that is thought to be the driver for most of the patterns seen in *P. interpunctella* population data (Bjørnstad et al., 1998; Sait et al., 1994). When resources are scarce the larger 4th and 5th instar larvae not only outcompete smaller larvae, but will also cannibalize eggs, smaller larvae, each other and pupae (Cameron et al., 2007; Reed, 1998). At 30°C, *P. interpunctella* egg‐to‐adult survival is reduced and overall fecundity is lower (Laughton et al., 2017; Parrett & Knell, 2018). This reduction in recruitment means that competition between larvae is likely to be reduced, which is consistent with the more even age distributions of larvae found at 30°C. Reduced larval competition is also consistent with the dramatic and, at first sight, counterintuitive increase in adult populations under temperature stress at 30°C. A series of studies have shown that if competition is over‐compensating, then an increase in mortality or a decrease in recruitment can lead to an apparently paradoxical increase in population size if it occurs at a life stage prior to competition acting (reviewed in (Schröder, Leeuwen, & Cameron, 2014). In the present case, it appears that one consequence of this is that a greater number of larvae reach adulthood.

Our predictions about the effects of temperature and diet quality on the generation cycling typical of *P. interpunctella* populations are not consistent with the patterns we found. In particular, poor diet here seems to be associated with a greater likelihood of generation cycling, and there is no indication of a reduction in cycle period with higher temperatures. Strikingly, generation cycling was lost in the 30°C, standard diet treatments but not in the poor diet populations at the same temperature. Modeling suggests that more asymmetric competition and higher fecundity are both associated with a greater tendency to display generation cycles (Briggs et al., 2000). Reduced fecundity and increased mortality at 30°C will reduce the degree of competition between larvae and make generation cycling less likely. We suggest that the increased resources available in the standard diet treatment reinforce this effect by further decreasing competition, leading to the lack of generation cycling seen in these populations. The more even distributions of larval ages seen in the 30°C populations is consistent with this, although the distribution for larvae in the poor diet treatment, which did show generation cycling, is similar to that for the standard diet treatment at this temperature, suggesting a possible further effect of food quality which we do not currently fully understand. One possible way for food quality to affect dynamics arises from the effects of egg cannibalism on population behavior. This was found to be necessary for generation length cycling in the models of Briggs et al. (2000) and in the absence of egg cannibalism age‐structured models of *P. interpunctella* dynamics predict half‐generation length cycles. Lower recruitment and abundant resources in the 30°C, standard diet populations could cause a reduction in egg cannibalism, reducing the likelihood of generation cycles and possibly accounting for the transient shorter period cycles seen in these cases.

The maintenance of 6‐week cycles in the 30°C, poor diet populations and also in the one 33°C population which cycled is intriguing, and given the reduction in development time and adult lifespan associated with higher temperatures (Laughton et al., 2017), it seems counterintuitive. A partial solution may lie in the fecundity schedules of the adult moths, which are similar at 27 and 30°C, with most females laying the majority of their eggs in the first few days of adulthood (Laughton et al., 2017). This means that the effective generation time might be more similar at these temperatures than would otherwise be thought. Alternatively, it is possible that the poor diet associated with generation cycles at both higher temperatures could lead to extended fecundity periods and longer adult lives. Reports are mixed regarding dietary effects on development time in *P. interpunctella* (Littlefair, Nunn, & Knell, 2016; McVean, Sait, Thompson, & Begon, 2002b), with McVean et al. reporting a small, nonsignificant increase in time from egg to eclosion in animals raised on poorer diet, whereas Littlefair et al. found that high levels of cellulose in a synthetic diet (i.e., a less nutritious diet) were associated with faster development. However, more cellulose was also strongly associated with longer adult lives in *P. interpunctella* (Littlefair et al., 2016). If this extended adult lifespan also leads to a longer period during which eggs are laid, this could compensate for shorter development associated with temperature and maintain cycle length at 6 weeks.

Long‐period population cycles have been reported from *P. interpunctella* populations before, but only with natural enemies present: The presence of both a granulosis virus and the parasitoid *Venturia canescens* leads to multigenerational cycles of 3–4 generations (Begon et al., 1996). This behavior was ascribed by the authors to the reduction in asymmetrical larval competition caused by these natural enemies, which seems to dampen generation–length cycles and lead to more classical predator–prey type cycles. More recently, Boots et al. (2009) showed that the granulosis virus could cause multigenerational cycles, but only when the animals were reared in a food with a high viscosity, causing increased spatial structure in the *P. interpunctella* population. In this case, increased local transmission between larvae of similar ages arising because of the decreased mixing in the population seems to have been driving the switch from single‐generation to multigeneration cycles. The long‐period cycles which appear to be evident in our data, by contrast, are not associated with the presence of any natural enemies, and there is no convincing pattern regarding the different food or temperature treatments. Many of our populations appear to show a period of low population sizes and small fluctuations between approximately 42 and 48 weeks, which could possibly be caused by some exogenous mechanism, although there is nothing in our records to indicate anything untoward during this time. This low period in the middle of the experiment could potentially account for the apparent periodicity showing up in the spectral analyses; hence, we are cautious in interpreting these long‐period cycles as evidence of cyclic behavior, especially when we consider that no other *P. interpunctella* single species population experiments have shown similar patterns.

Many economically important species of insects and fish display generation cycles similar to those found in laboratory populations of *P. interpunctella*. While we do not have enough knowledge of their temperature relationships to make specific predictions, it is likely that a moderate amount of warming will also cause some of these to display similarly altered population dynamics. At a minimum, such changes would alter harvest, management, and control requirements, and they could, potentially, cause severe impacts on agriculture and fisheries. More generally, age structure is a crucially important factor in the population ecology of the majority of animal species (Begon, Townsend, & Harper, 2005), and these data demonstrate that moderate warming can alter age structures considerably, with important consequences for population structures and behavior. Understanding the effects of climate change at a population level is crucial for conservationists, pest control practitioners, and natural resource managers and this study makes clear the necessity of in depth, early research in order to predict what these effects will be.

## CONFLICT OF INTERESTS

The authors declare that they have no competing interests.

## AUTHORS' CONTRIBUTIONS

RK and AL designed the study. AL was responsible for the practical work and data collection. RK analyzed the data. RK wrote the first draft of the manuscript, and AL and RK revised it and produced the final version.

## Figures and Tables

**Figure 7:**
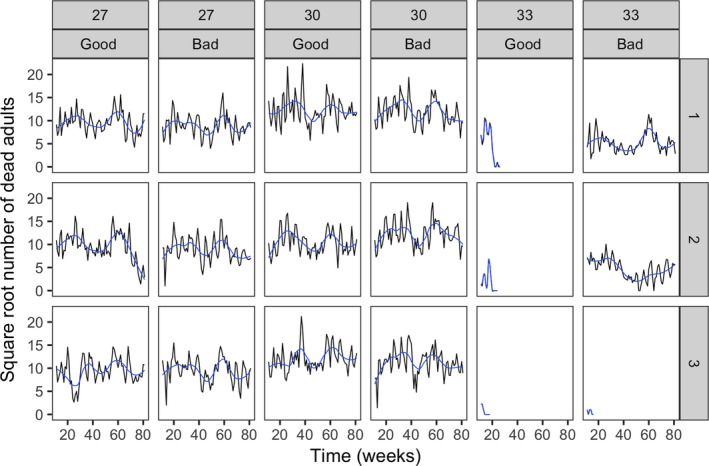
The 18 population time series, square root transformed, with a fitted Loess smoother which makes the long‐period cycles clear

## Data Availability

The datasets used here are available from the Dryad repository at https://doi.org/10.5061/dryad.4fg24s2, along with a file containing the R code for all of the analyses and data visualizations presented here.

## APPENDIX 1

### ANALYSIS OF ADULT POPULATION SIZES

Figure 2a (main text) shows the mean adult population sizes for each population. We can fit a model to these data to assess effect sizes and the statistical significance of the effects of temperature and food quality. Because most of the high temperature populations did not persist, we will restrict the analysis to the 27 and 30° treatments only.

#### Minimal model

Model selection by removal of nonsignificant terms leaves us with only temperature as an explanatory variable.

Since food quality has no significant effect we have a few more *df* to play with so we can include the two persistent populations at 33° as well

Overall, temperature has a substantial effect on the mean population sizes. Increasing the temperature from 27 to 30° increases mean population size roughly 1.5 times, but going from 27° to 33° means that those populations manage to persist at the higher temperature have a mean size which is roughly a third of the 27° populations and a fifth of the 30° ones.

### VARIABILITY OF ADULT POPULATIONS

Figure 6 shows the coefficients of variability for the adult population trajectories for each population. 30° populations were slightly less variable than 27° populations. The 33° populations that persisted had much higher CVs, but this is possibly more of an effect of their low mean population size than anything else.

Can we discern any statistically significant patterns in the variance of the adult populations?

No significant interaction term and no effect of food. We can include the 33° populations in a final model.

CVs are significantly lower in the 30° populations and then significantly raised in the 33° populations. There is no discernable effect of food quality.

### LARVAE

Figure 2b (main text) shows the mean counts per week for the total number of larvae per food section. By contrast with the data for adults, there were fewer larvae in the 30° treatment than in the 27° one. The two 33° treatments which persisted had very low numbers.

Analysis of these data is similar to that for the adult populations. An initial model fitted to the 27 and 30° data only is reduced to one with only temperature as an explanatory variable and we can add in the two persisting 33° populations to that to give a final minimal adequate model with temperature as an explanatory variable but not food quality. By contrast with the adult populations, larval populations are smaller in the 30° treatment than in the 27° one, but as with the adult populations the two 33° populations which persisted have very low larval counts.

### AGE STRUCTURE OF THE LARVAL POPULATION

Time‐averaged proportions of 3rd, 4th, and 5th instar larvae are shown in Figure 3 (main text). There are clear differences between treatments, with the highest proportions of 5th instar larvae in the 27 degree good food populations and the lowest proportions being in the 30° populations.

These differences in the proportions are statistically significant. They can be analyzed using a linear model with the mean number in one stadium (either 5th or 3rd instar larvae, it makes little difference) as a response variable and with the total number of larvae as a covariate.

Alternatively, an analysis using the proportion of 5th instar larvae as the response:

The age structures of the larval populations are therefore significantly different between treatments, with a significant interaction between temperature and food quality. The 27°, good food populations have the most 5th instar larvae, and either increasing the temperature or changing to poor food decreases the relative abundance of 5th instar larvae in favor of younger animals. Food quality has little effect on age structure for the 30° populations, however, all of which have roughly similar age structures.

### TIME SERIES ANALYSIS

#### Long‐period cycles

The smoothed spectral density plots show that in fact there are two important periodicities in the data—firstly a long‐period cycle of between 35 and 25 weeks, as indicated by the peak on the left hand side of the graphs, and secondly short‐period generation cycles of 6 weeks overlaid onto the longer term cycles. This is shown by the peak that in most cases aligns with the vertical dotted line, which indicates a frequency of 1/6 on each graph.

These long‐period cycles are visualized in Figure 7. Some populations show longer period cycles (approx. 35 weeks) and some shorter (approx 25 weeks). Note that because of the length of these cycles the spectral analysis will only fit peaks corresponding to periods of 72,36,24,18, etc., so the precise lengths of the long‐period cycles are hard to estimate.

There is no apparent association between temperature and cycle length, but there is between food quality and cycle length. If we consider the 27 and 30° populations, all the poor food populations show short cycles (six out of six), whereas most of the good food populations show longer cycles (4 out of 6). This is not quite statistically significant:
